# Effects of Reduced Graphene Oxides on Apoptosis and Cell Cycle of Glioblastoma Multiforme

**DOI:** 10.3390/ijms19123939

**Published:** 2018-12-07

**Authors:** Jaroslaw Szczepaniak, Barbara Strojny, Ewa Sawosz Chwalibog, Slawomir Jaworski, Joanna Jagiello, Magdalena Winkowska, Maciej Szmidt, Mateusz Wierzbicki, Malwina Sosnowska, Jasmina Balaban, Anna Winnicka, Ludwika Lipinska, Olga Witkowska Pilaszewicz, Marta Grodzik

**Affiliations:** 1 Department of Animal Nutrition and Biotechnology, Faculty of Animal Sciences, Warsaw University of Life Sciences, 02-787 Warsaw, Poland; jaroslaw_szczepaniak@sggw.pl (J.S.); barbara_strojny@sggw.pl (B.S.); ewa_sawosz@sggw.pl (E.S.C.); slawomir_jaworski@sggw.pl (S.J.); mateusz_wierzbicki@sggw.pl (M.W.); malwina_sosnowska@sggw.pl (M.S.); jasmina_balaban@sggw.pl (J.B.); 2 Department of Chemical Synthesis and Flake Graphene, Institute of Electronic Materials Technology, 01-919 Warsaw, Poland; joanna_jagiello@itme.edu.pl (J.J.); magdalena.winkowska@itme.edu.pl (M.W.); ludwika_lipinska@itme.edu.pl (L.L.); 3 Department of Morphologic Sciences, Faculty of Veterinary Medicine, Warsaw University of Life Sciences, 02-787 Warsaw, Poland; Maciej.Szmidt@Acurian.com; 4 Department of Pathology and Veterinary Diagnostics, Faculty of Veterinary Medicine, Warsaw University of Life Sciences, 02-787 Warsaw, Poland; anna_winnicka@sggw.pl (A.W.); olga_witkowska_pilaszewicz@sggw.pl (O.W.P.)

**Keywords:** reduced graphene oxide, brain tumor, glioblastoma multiforme, JC-1, cell cycle, apoptosis

## Abstract

Graphene (GN) and its derivatives (rGOs) show anticancer properties in glioblastoma multiforme (GBM) cells in vitro and in tumors in vivo. We compared the anti-tumor effects of rGOs with different oxygen contents with those of GN, and determined the characteristics of rGOs useful in anti-glioblastoma therapy using the U87 glioblastoma line. GN/ExF, rGO/Term, rGO/ATS, and rGO/TUD were structurally analysed via transmission electron microscopy, Raman spectroscopy, FTIR, and AFM. Zeta potential, oxygen content, and electrical resistance were determined. We analyzed the viability, metabolic activity, apoptosis, mitochondrial membrane potential, and cell cycle. Caspase- and mitochondrial-dependent apoptotic pathways were investigated by analyzing gene expression. rGO/TUD induced the greatest decrease in the metabolic activity of U87 cells. rGO/Term induced the highest level of apoptosis compared with that induced by GN/ExF. rGO/ATS induced a greater decrease in mitochondrial membrane potential than GN/ExF. No significant changes were observed in the cytometric study of the cell cycle. The effectiveness of these graphene derivatives was related to the presence of oxygen-containing functional groups and electron clouds. Their cytotoxicity mechanism may involve electron clouds, which are smaller in rGOs, decreasing their cytotoxic effect. Overall, cytotoxic activity involved depolarization of the mitochondrial membrane potential and the induction of apoptosis in U87 glioblastoma cells.

## 1. Introduction

In 2004, a group of scientists led by Geim and Novoselov discovered stable free graphene, a material previously known only in theory. Geim and Novoselov obtained a graphene layer via mechanical exfoliation [1]. This marked the beginning of the “graphene era”, which continues to this day. Research has been focused on creating new methods for the production, modification, and advantageous application of this material in the technical, biological, and medical fields.

Reduced graphene oxide (rGO) and graphene oxide (GO) are derivatives of graphene (GN). Graphene has a perfect crystal lattice structure, sp^2^ hybridization, and numerous useful properties. These properties include a high electrical conductivity, inherent mechanical strength, and the ability to absorb white light. In comparison, GO is highly hydrophilic due to the presence of oxygen groups and therefore can form stable aqueous suspension. rGO has more oxygen groups than GN, but less than GO. Therefore, rGO is less hydrophilic than GO, but also has a higher electrical conductivity. [1,2].

In addition to the numerous applications of these materials in electronics, energetics, sensors, and filtration, graphene plates and their derivatives are also being assessed for use in medicine, such as in cancer treatment [3,4]. Glioblastoma multiforme (GBM) has an astrocytic origin and is a malignant primary tumor of the brain. It represents the most commonly occurring cancer of the central nervous system and is usually grade IV histologically. The most important features of this type of cancer are the presence of necrotic areas and characteristic vascularization (numerous microvessels and glomerular vessels), as well as the presence of atypical cells, nuclear pleomorphism, and high proliferative activity of malignant cells [5]. The type and place of glioblastoma growth render this tumor incurable. The average incidence rate of GBM is 3.19 per 100,000 persons per year, and the average overall survival after diagnosis is approximately 15 months [6]. Our previous study has shown that graphene and its derivatives can be cytotoxic to glioblastoma cells in vitro and in vivo. Upon entering, GN, GO, and rGO induced apoptosis, and reduced cell viability and proliferation, in the U87 and U118 glioblastoma cell lines. GN, GO, and rGO also reduced the mass and volume of tumors cultured on chorioallantoic membranes. However, these compounds differ in strength; the most active is GN, the next is rGO, and the least active is GO [7,8]. Moreover, depending on the method of production, graphene-derived materials can be modified to have a wide range of characteristics, including different sized flakes, numbers and types of functional groups, numbers of defects, and hydrophobicity and electrical properties [9].

Graphene-based materials are expected to lead a revolution in medicine, but they are also associated with difficulties such as protein corona formation, the EPR (Enhanced Permeability and Retention) effect, and biodistribution [10]. In biological environments, graphene flakes can interact with the components of the physiological medium, which may induce radical changes in the size, shape, or chemistry of the surface flakes. Within the blood stream, the flakes can interact with cell membranes and induce reactive oxygen species, resulting in haemolysis [11]. It has been shown that rGO induced a toxic effect on endothelial cells [12]. The location of the glioma means that this tumor is treated in a different way than is traditional, like intravenously or intra-arterially. Active substances can be efficiently used with direct stereotactic injections into the tumor, or by using biomaterials supersaturated with these molecules, which intraoperative are located in a lodge after tumor reception [13]. Unconventional drug delivery causes the most common difficulties, in which nanomaterials distribution does not occur.

In the present study, we characterized three forms of rGO plates prepared from the same GO. We also investigated whether materials included in the rGO groups exert the same biological effects on glioblastoma multiforme cells, and how these effects compare with those of pure graphene.

We hypothesized that rGO does not exert identical effects on cells, but that the anti-cancer effect is likely greater compared with that of pure graphene. Effectiveness was examined by assessing viability, metabolic activity, cell cycle dynamics, and level of apoptosis. In the final stage of analysis, we examined gene expression involved in apoptosis and the cell cycle. Using these results, we sought to determine whether it is possible to identify the characteristics of rGO flakes that may be useful in anti-glioblastoma therapy. Our results indicate that it is possible to determine which pathway of apoptosis activation occurs in cells treated with these materials, and whether this same pathway is induced by all the examined materials.

## 2. Results and Discussion

In this study, we aimed to determine the cytotoxicity of pure graphene flakes (GN) and compare it with that of reduced graphene oxide flakes (rGO) produced by various methods. We also aimed to determine the pathway of cell death induced by the administration of these materials, and whether it is associated with a loss of mitochondrial membrane potential or with blocking the individual phases of the cell cycle.

### 2.1. Physicochemical Characterization of GN and rGO Flakes

The same graphene oxide (GO) was used as the primary material for the production of reduced graphene oxides via different chemical methods. The use of the same primary material does not lead to the formation of identical products. Therefore, physicochemical analyses (Raman radiation scattering, analysis of the morphology of the materials via transmission electron microscopy, elemental analysis, resistivity measurements, and measurements of zeta potential) were performed to examine the characteristics of the derived products.

**Figure 1:**
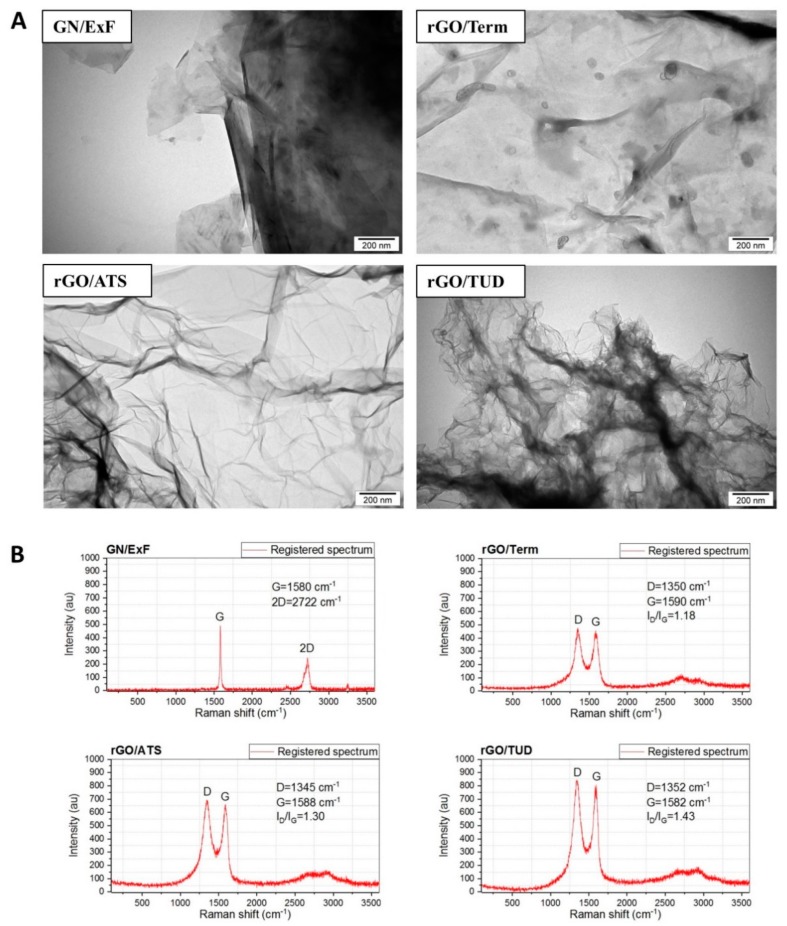
Transmission electron microscopy (TEM) images and Raman spectra of GN and rGO flakes. (**A**) Images of GN/ExF flakes and three types of rGO (rGO/ATS, rGO/Term, and rGO/TUD). (**B**) Spectra consist of three bands: D band located at ~1350 cm^−1^, G band at ~1580 cm^−1^, and 2D band at ~2700 cm^−1^. G band is characteristic of each graphene sample. The appearance of the D-band is typical of rGO samples because some oxygen functional groups remain after the reduction of GO. A strong 2D band only appears for graphene (GN); for rGOs, the 2D band occurs as a weak and broad signal. **Abbreviations**: rGO, reduced graphene oxide; GN, graphene.

The quality of the prepared materials was examined using Raman spectroscopy. This is based on analysis of the three main peaks in the spectra of graphene and reduced graphene oxides, as well as the peak position, peak width, and relative intensity ratio (I_D_/I_G_). These main bands are designated as D, G, and 2D, and are centered at 1350 cm^−1^, 1580 cm^−1^, and 2680 cm^−1^, respectively [14]. The results of Raman spectroscopic analysis (Figure 1) confirmed that GN/ExF belonged to graphene, and rGO/Term, rGO/ATS, and rGO/TUD belonged to reduced graphene oxides. The incorporation of GN/ExF graphene into graphene materials was confirmed based on existing data from the literature; G and 2D bands were observed at 1580 cm^−1^ and 2700 cm^−1^, respectively. rGO/Term, rGO/ATS, and rGO/TUD were confirmed as reduced graphene oxides based on D and G bands appearing at 1350 cm^−1^ and 1580 cm^−1^, respectively (also based on existing data in the literature). The G-band in Raman spectra corresponds to the C-C chains of carbon atoms. The D-band is correlated with structural defects, such as holes, rotated carbon bonds, or deformed rings, and with chemical defects corresponding to oxy-functional groups (carbonyl and hydroxyl) attached to the planar carbon lattice and edges. When the oxygen content is decreased, which results from the more efficient reduction of graphene oxide, the intensity ratio I_D_/I_G_ increases. This can correspond to the appearance of numerous (but small) restored sp^2^ regions. The size of defect-free sp^2^ regions is inversely related to the ratio of D band intensity to G band intensity (I_D_/I_G_), and to the full width at half-maximum (FWHM) of the G band [15,16]. Based on the G band width and I_D_/I_G_ ratio, one can assume that the sp^2^ clusters grow larger and the ID/IG increases, while the G band becomes narrower, than those in the initial GO (79 cm^−1^): rGO/Term 71.96 cm^−1^, rGO/ATS 68.84 cm^−1^, and rGO/TUD 59.18 cm^−1^ [17,18,19]. These results are shown in Figure 1.

Moreover, Raman measurement can provide information about the number of graphene layers. It can be estimated after 2D peak deconvolution, its shape and intensity (compared to the intensity of G peak) analysis. However, it cannot be used for GO and rGO materials, but for less defected graphene (e.g., exfoliated graphite or CVD graphene) [14]. The Raman spectrum of GN/ExF indicates that this graphene-like material consists of about 5–10 layers as the 2D peak can be split into two peaks and its intensity is two times lower than that of the G peak.

**Figure 2:**
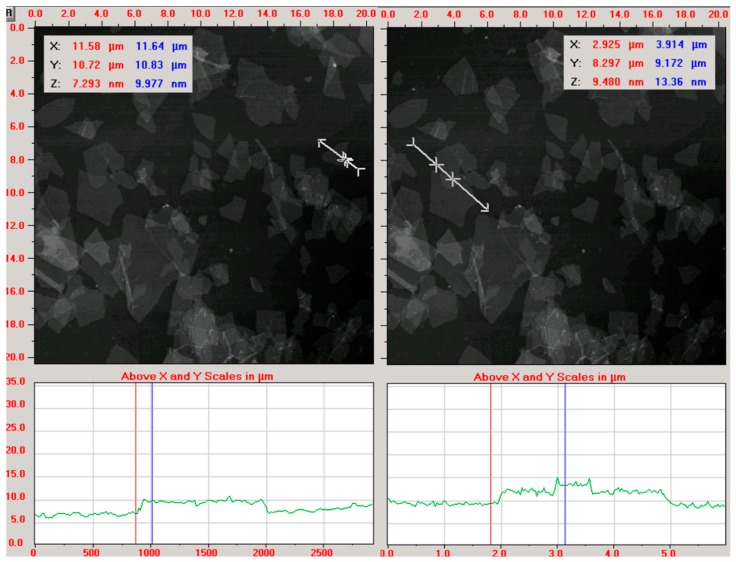
AFM measurement of GO flakes. X and Y scales are in μm and Z scale is in nm. Z axis corresponds to GO flake thickness. Left profile shows the thickness of a single GO flake (2.7 nm) and the right shows the GO thickness of a single flake (2.5 nm) and two flakes that overlap (3.4 nm). **Abbreviations**: AFM, atomic force microscope; GO, graphene oxide.

Three samples of rGO were prepared by the reduction of GO, so the number of layers of these materials is the same as that of GO flakes. Due to a strong tendency to agglomeration of rGO flakes, AFM measurements were only conducted for starting material (GO). The AFM measurement is shown in Figure 2. The thickness of GO flakes is about 2.5–2.7 nm, which corresponds to two-layer GO flakes.

Three samples of rGO were prepared by the reduction of GO, so the number of layers of these materials is the same as that of GO flakes. Due to a strong tendency to agglomeration of rGO flakes, AFM measurements were only conducted for starting material (GO). The AFM measurement is shown in Figure 2. The thickness of GO flakes is about 2.5–2.7 nm, which corresponds to two-layer GO flakes.

TEM analysis was used to assess the morphology of the flakes of rGO and GN. Figure 1A shows the representative microscopic images. In reduced graphene oxides (rGO/ATS and rGO/TUD), strong corrugation of the imaged flakes was visible. Oh and Zhang also confirmed that graphene and reduced graphene oxides most often occur in the form of more or less corrugated, single, or multilayer flakes [20]. The shapes of the flakes of GN and rGO were irregular, and their edges were often jagged. All of the graphene derivatives investigated in this study were able to agglomerate. Suparut and Somchai confirmed that nanosheets of graphene are similar to wavy veils, rippling and entangling with each other. They are transparent and stable under an electron beam, which is used to confirm the existence of two-dimensional graphene nanosheets [21]. In the TEM image, dark areas indicated the dense nanostructure of several graphene layers or reduced graphene oxides. Higher areas of transparency indicated a much thinner layer, which, in the case of reduced graphene oxides, depends on the degree of reduction. Lower oxygen improves delamination of the material [22]. We observed this in our rGO/ATS flakes by comparing them to rGO/TUD, in which strong corrugation and overlap was visible.

Elemental analysis (Table 1) showed a low oxygen level (3%) in the reduced graphene oxide rGO/ATS; this may have resulted from the high sulfur content of the reducer used (ammonium thiosulphate). At approximately 1%, the reduced rGO/Term graphene oxide showed the lowest percentage of oxygen. However, it was very challenging to measure resistance for this rGO due to the difficulty of compressing the sample. The highest oxygen content of approximately 16% was observed in rGO/TUD. Resistivity (Rs) was 21 Ohm/square and 142 Ohm/square for rGO/ATS and rGO/TUD, respectively, and 0 Ohm/square for GN/ExF. Flakes with a lower oxygen content also have a lower resistivity, thereby serving as better conductors of electricity.

The results of resistivity measurements are not shown for GN/ExF because the sample quantity was too scant, and the measurement performance was relatively low. Results of resistivity measurements are also not shown for rGO/Term because we were unable to compress the sample for measurement. This is a consequence of highly reduced GO, where no hydrogen bonds were present in the flakes. The GN/ExF sample is pure graphene, which does not contain oxygen atoms; hence it was excluded from elemental analysis.

**Chart A1:**
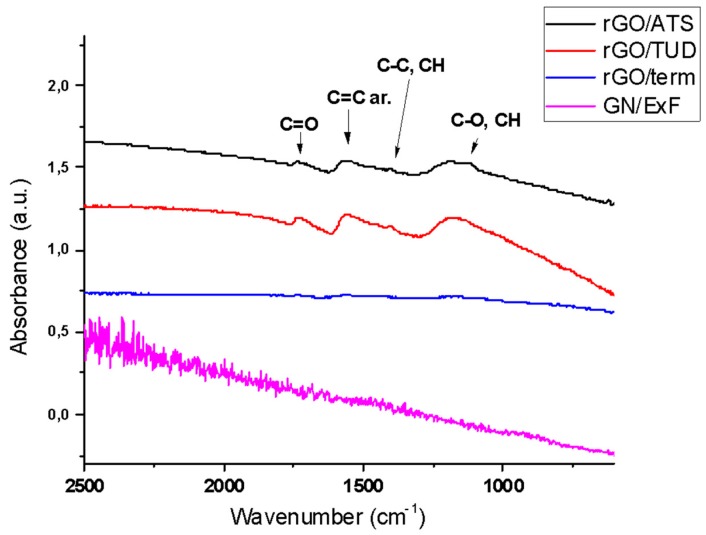
FTIR spectra of four graphene-like samples. Band at 1735 cm^−1^ refers to C=O modes of carbonyl groups. Peak that appears at around 1560 cm^−1^ indicates C=C bonding in aromatic tructure of the materials. In the range of 1400 cm^−1^ and 1450 cm^−1^ there are overlapped bands corresponding to CH vibrations as well as to C-C (stretching) in aromatic structure and to OH groups. Peaks at around 1000–1200 cm^−1^ correspond to C-O (stretching) and CH (aromatic) bonds. **Abbreviations**: FTIR, fourier transform infrared spectroscopy.

Elemental analysis does not provide information about specific oxygen groups, so we conducted FTIR measurements (Chart A1). The FTIR spectra of rGO/TUD and rGO/ATS are quite similar, suggesting the existence of nearly the same functional groups. The band at 1735 cm^−1^ refers to C=O modes of carbonyl groups. The peak that appears at around 1560 cm^−1^ indicates C=C bonding in the aromatic structure of the materials. In the range of 1400 cm^−1^ and 1450 cm^−1^, there are overlapped bands corresponding to CH vibrations, as well as to C-C (stretching) in the aromatic structure and to OH groups. Peaks at around 1000–1200 cm^−1^ correspond to C-O (stretching) and CH (aromatic) bonds. No clearly seen peaks in GN/ExF and rGO/Term spectra suggest that there are no oxygen functional groups in the structure of these samples. This corresponds to elemental analysis, where these materials were found to have 0 and 1% oxygen content, respectively.

**Chart 1:**
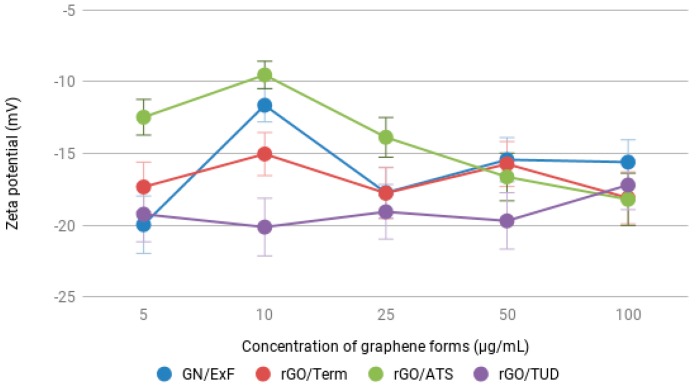
Zeta potential of GN and rGOs. Zeta potential is expressed in mV and was measured at different concentrations (5, 10, 25, 50, and 100 µg/mL) of the tested GN and rGOs. **Abbreviations**: mV, millivolt; GN, graphene; rGO, reduced graphene oxide.

Zeta potential (Chart 1) was analyzed to characterize the surface charge and stability of the tested suspensions. The more oxygen-containing functional groups that exist on the surface of the rGOs, the higher the stability of the solution [23]. Zeta potentials were measured using five different concentrations of GN and each rGO. At concentrations of 5 and 10 μg/mL, we observed the highest divergence between the flakes of the GN group and rGO groups. rGO/ATS showed the lowest stability (−9.51 mV) at a concentration of 10 μg/mL; rGO/TUD showed the highest stability (−20.13 mV) at the concentration of 10 μg/mL. At concentrations of 50 and 100 μg/mL, all studied materials were characterized as flakes having a similar stability. Minidvan (2016) showed that the zeta potential of their rGO was −3.81 mV; this was due to a decreased amount of oxygen-containing functional groups compared with those of GO [24]. Elemental analysis, conducted on rGO/TUD, showed that it had the highest percentage of oxygen, thus forming the most stable suspension observed in our study. GO easily disperses in the hydrophilic environment. To restore the sp2 carbon structure of graphene sheets, reduction is necessary. As a result of the reduction, graphene sheets contain less functional groups and consequently more sp2 hybridized carbon atoms, what is also directly connected with a higher number of delocalized electrons. rGO has a tendency to agglomerate, lowering the colloidal stability of the graphene flakes at the same time [25]. Thereby, the higher the degree of the reduction, the lower the stability, which is clearly visible in rGO/TUD (higher stability) and rGO/Term (lower stability) samples. In a low concentration of the hydrocolloids (especially 10 µg/mL) of highly reduced samples, such as rGO/ATS and GN/ExF, a decrease of the stability and increase of the surface charge are observed at the same time. This is due to weaker interactions between rGO flakes and stronger interactions between rGO and the polar dispersant, which is water. In the rGO/ATS sample, in the lowest concentration (5 µg/mL), the hydrocolloid is stable because of a greater distance between flakes, and in the 10 µg/mL concentration, the distance between flakes is reduced and the flakes start to form agglomerates, which do not drop to the bottom; therefore, the stability decreases. In higher concentrations, flakes agglomerate and they drop to the bottom of a tube, resulting in the reduction of the hydrocolloid concentration and thus a higher stability of the colloid. As a consequence, the stability of the colloid is similar to the rGO/ATS sample at a 5 µg/mL concentration. In the least reduced sample, rGO/TUD, the highest stability is observed in all concentrations.

The hydrodynamic diameter of the flakes and the dispersibility of the suspension were estimated using ZetaSizer. Detailed results are shown in Table 1. The hydrodynamic diameter of the flakes fluctuated within 1–5 μm, and flake suspensions were polydispersed. By polydispersity, we mean the heterogeneity of the flake suspension. This phenomenon was found based on measurements of the hydrodynamic diameter; the Polydispersity Index (PI) was calculated by the ZetaSizer measurement software. From each type of material, we tested three samples, and each sample was measured 10 times. In magnification 100,000× in TEM, we observed the presence of different sized flakes (from few nm to few μm). In this way, we confirmed the polydispersity index calculated from ZetaSize.

### 2.2. The Influence of GN and rGO on Cellular Morphology and Viability

**Figure 3:**
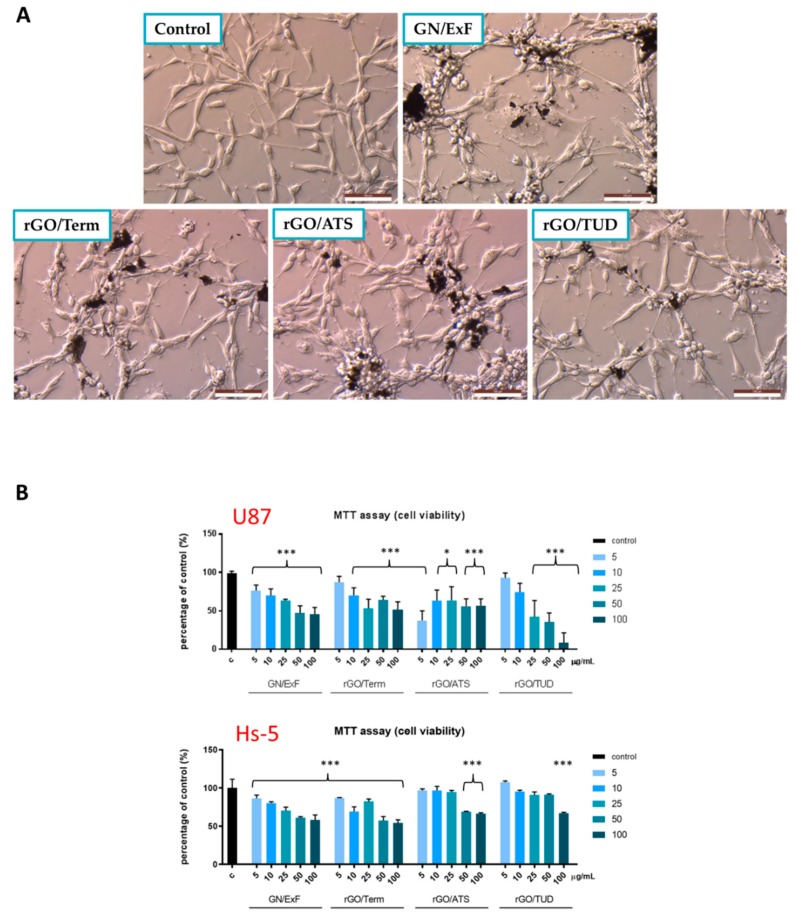
Morphology and cell viability of untreated U87 cells, and those treated with GN and rGO flakes, as evaluated by optical microscopy and viability assays. (**A**) The images show untreated U87 cells and those treated with GN and rGO flakes. Control cells were not treated with graphene flakes. Scale bar: 100 µm. Cell viability was determined using the (**B**) MTT assay. U87 and Hs-5 cells were exposed to GN and rGO flakes at concentrations of 5, 10, 25, 50, and 100 μg/mL for 24 h. Values are expressed as mean ± standard deviation. Statistical significance between the control and treated cells is indicated by an asterisk and was assessed using Bonferroni’s multiple comparison test. Differences with *p*-value < 0.05 were considered significant. One asterisk (*) *p*-value < 0.01, three asterisks (***) all with *p*-value < 0.001. **Abbreviations**: rGO, reduced graphene oxide; GN, graphene; MTT, 3-(4,5-dimethylthiazol-2-yl)-2,5-diphenyltetrazolium bromide.

We assessed the interactions of rGO flakes, compared with those of GN, with glioblastoma cells. For this, we examined how cytotoxicity of the oxygen content in rGOs exerts different effects on glioblastoma cells. First, we assessed the effect of graphene flakes on the general morphology of glioblastoma cells (examined via light and confocal microscopy), and then we examined the viability and metabolic activity of the cells using an MTT assay. An MTT assay was also performed with normal cell line Hs-5. In the control group (untreated U87 cells), examined using light microscopy, the cells showed a clearly outlined cellular body with long protrusions, and the tendency to grow in groups towards a spheroid formation without forming a typical monolayer (Figure 3A). Cell nuclei were clearly outlined with visible nucleoli.

**Figure A1:**
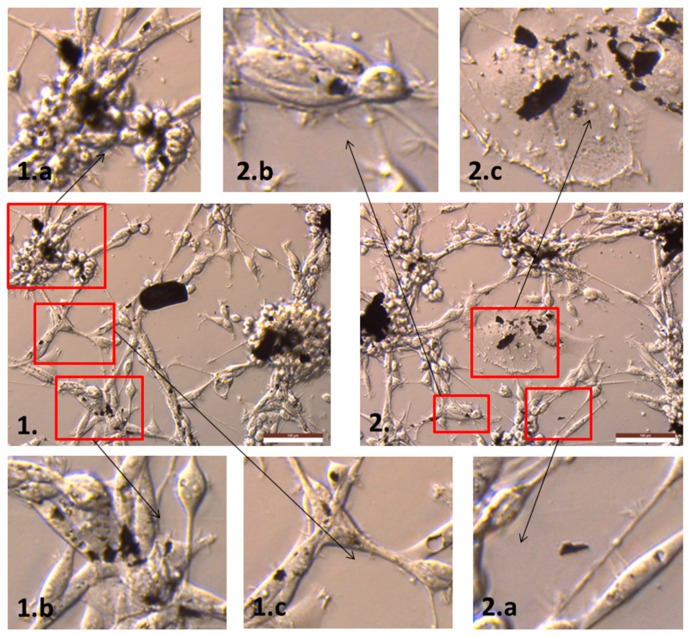
Morphology of U87 cells treated with GN flakes, as evaluated by optical microscopy. Cells treated with graphene flakes (at concentration of 50 µg/mL)—(1–2) GN/ExF. Regions of interest marked with red borders, are shown enlarged, and are indicated by black arrows. Scale bar: 100 µm.

**Figure A2:**
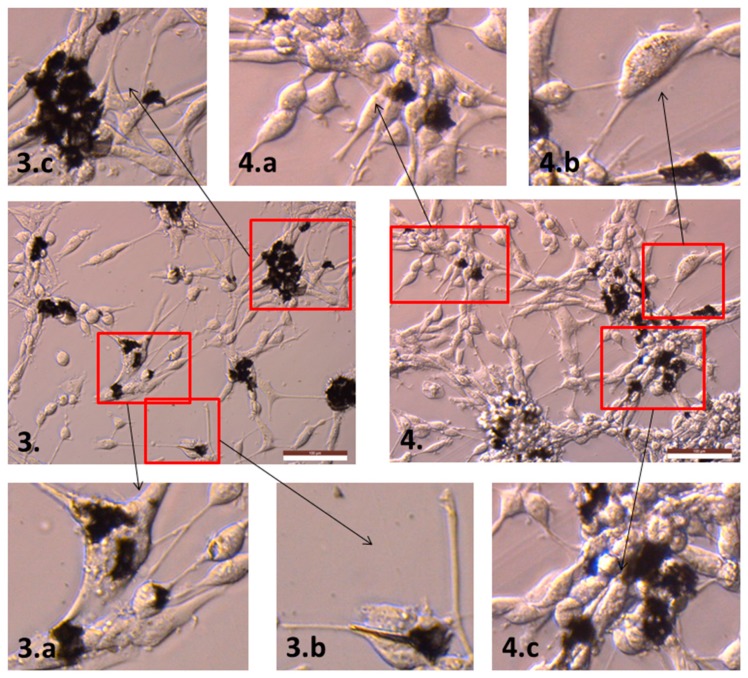
Morphology of U87 cells treated with rGO flakes, as evaluated by optical microscopy. Cells treated with graphene flakes (at concentration of 50 µg/mL)—(3–4) rGO/ATS. Regions of interest marked with red borders, are shown enlarged, and are indicated by black arrows. Scale bar: 100 µm.

**Figure A3:**
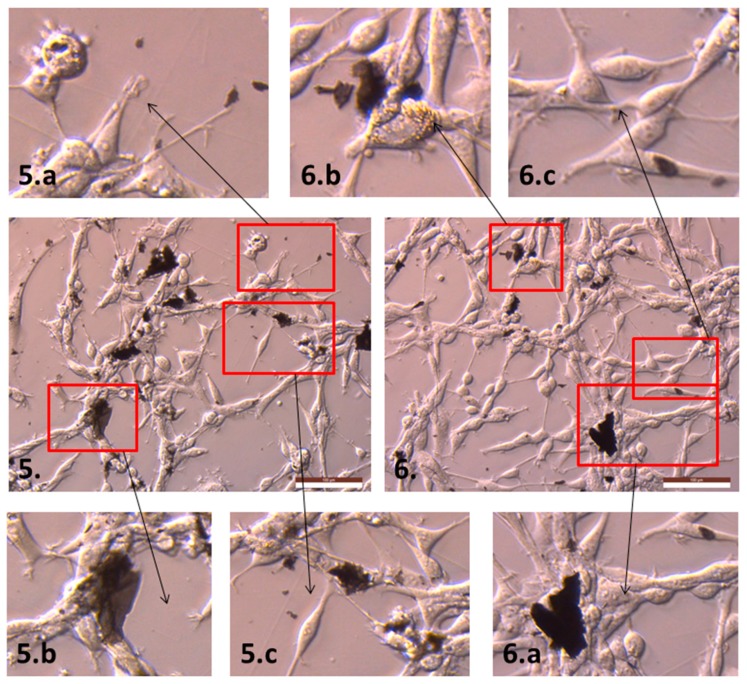
Morphology of U87 cells treated with rGO flakes, as evaluated by optical microscopy. Cells treated with graphene flakes (at concentration of 50 µg/mL)—(5–6) rGO/Term. Regions of interest marked with red borders, are shown enlarged, and are indicated by black arrows. Scale bar: 100 µm.

**Figure A4:**
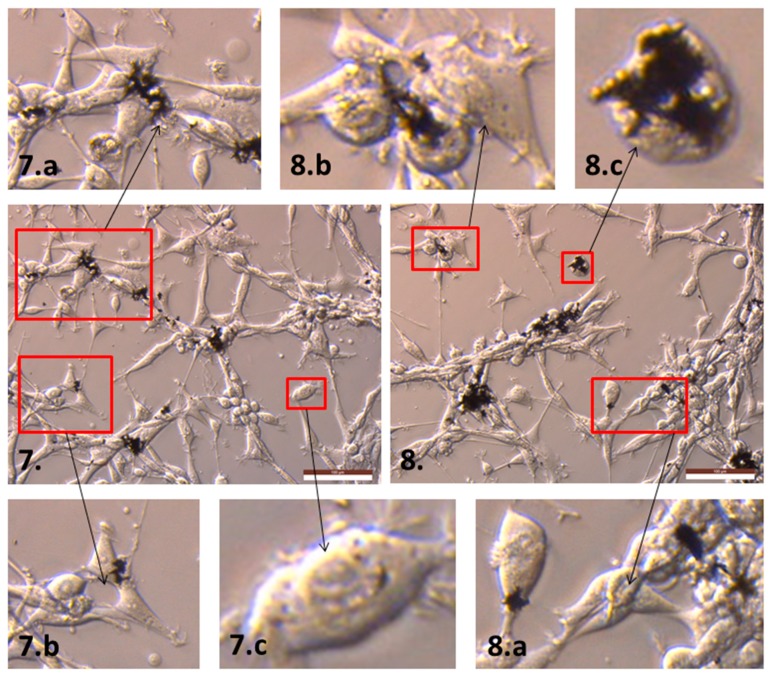
Morphology of U87 cells treated with rGO flakes, as evaluated by optical microscopy. Cells treated with graphene flakes (at concentration of 50 µg/mL)—(7–8) rGO/TUD. Regions of interest marked with red borders, are shown enlarged, and are indicated by black arrows. Scale bar: 100 µm.

In the examined samples, the number of cells in the field of view was kept constant (Figure 3A). In the samples treated with GN/ExF (Figure A1) and rGO/ATS (Figure A2, large groups of cells with well-adhered graphene flakes were clearly visible. This indicates that in these cases, graphene was able to form agglomerates. In most cases, graphenoid forms overlapped with cells. The most individual graphene flakes could be clearly seen in images treated with rGO/Term (Figure A3). Graphene flakes tended to adhere to the central part of the cellular body, such as the cytoplasm and nucleus, rather than to cellular protrusions. The greatest changes in cell morphology, such as pore formation called light spots [26], were observed in cells treated with GN/ExF and rGO/TUD (Figure A4). GN/ExF was the least compatible due to a lack of oxygen-containing functional groups. The images also showed more oval cell bodies in cells treated with rGO/ATS. Cells treated with GN/ExF did not show oval cell bodies, and were more elongated. Lammel and co-workers observed the impact of graphene oxide on the ultrastructure of the plasma membrane and found that nano-sized graphene penetrates the plasma membrane of the HepG2 cell line [27]. They showed that at the site of interaction between the graphene nanoplatelet and the plasma membrane, membrane invagination and some disruption of the plasma membrane occur. Other researchers reported that the exposure of cells to high concentrations of graphene decreased the membrane integrity in GLC-82, MCF-7, Panc-1, and MDA-MB-231 cell lines [28].

An evaluation of cell morphology using light and confocal microscopy showed that the effects observed in all the treated groups were similar. Treatment with rGO/ATS was the most effective against the glioblastoma line U87, causing complete destruction of the cell membrane. Based on the results of microscopic observations, we used the MTT assay to assess whether graphene and its derivatives also affect the viability and metabolic activity of the stromal Hs-5 and glioblastoma U87 cells. In the case of non-cancerous Hs-5 cells in GN/ExF and rGO/Term treated groups, they caused statistically significant changes in all tested concentrations, but a lower decrease in metabolic activity than in the case of U87 cells. For the rGO/ATS group, a statistically significant decrease in the viability of normal cells was observed at the concentrations of 50 and 100 µg/mL, whereas in the rGO/TUD group, this was only seen at the concentration of 100 µg/mL. In both groups, graphene flakes were found to be less toxic to healthy cells than cancerous U87 cells. For the U87 cell line MTT assay, a significant effect (*p*-value < 0.0001) was observed for each group (Figure 3B). The highest decrease in metabolic activity, at 8.69 ± 12.88%, was found in the group treated with rGO/TUD at a concentration of 100 μg/mL. Interestingly, in the group treated with rGO/ATS, the lowest viability, at 37.7 ± 12.55%, occurred at a concentration of 5 μg/mL rGO/ATS. In other groups treated with GN/ExF and rGO/Term, ~50% mortality was observed at concentrations ranging from 25 to 100 μg/mL. In a few cases, such as in the first group, the viability did not decrease below 72%, while the metabolic activity of the cells decreased up to 45% with the increasing concentration of GN/ExF. This may indicate a disturbance in the metabolic activity of the mitochondria with an intact cellular membrane. In the group treated with rGO/ATS, the lowest metabolic activity was observed at a concentration of 5 μg/mL. This may be due to the fact that in the lower concentration, the flakes are not agglomerated and, consequently, they can induce a stronger cytotoxic effect.

Because of their size, the flakes of GN/ExF, rGO/ATS, rGO/Term, and rGO/TUD have difficulty penetrating the cell; however, they physically or biologically reduce the viability of U87 cells. This may have been caused by a reduced amount of hydrophilic groups. The results confirm previous studies, indicating that GN and rGO flakes show significant toxicity against several cell lines (U251, U87, U118) [7,8,29,30]. rGO and GN can adhere to the two-layered lipid membrane due to hydrophobic interactions with the membrane [31]. Consequently, exposure to high concentrations of GN and rGO causes physical or biological damage to the cell membrane, accompanied by the destabilization of actin filaments and the cytoskeleton [32]. Moreover, one of our previous studies showed that functionalizing rGO with amino acids (such as proline) decreases the cytotoxic effects of graphene derivatives; this may have occurred because the amino acid blocked the hydrophobic of the molecules which strongly interact with the cellular membrane [33].

**Figure 4:**
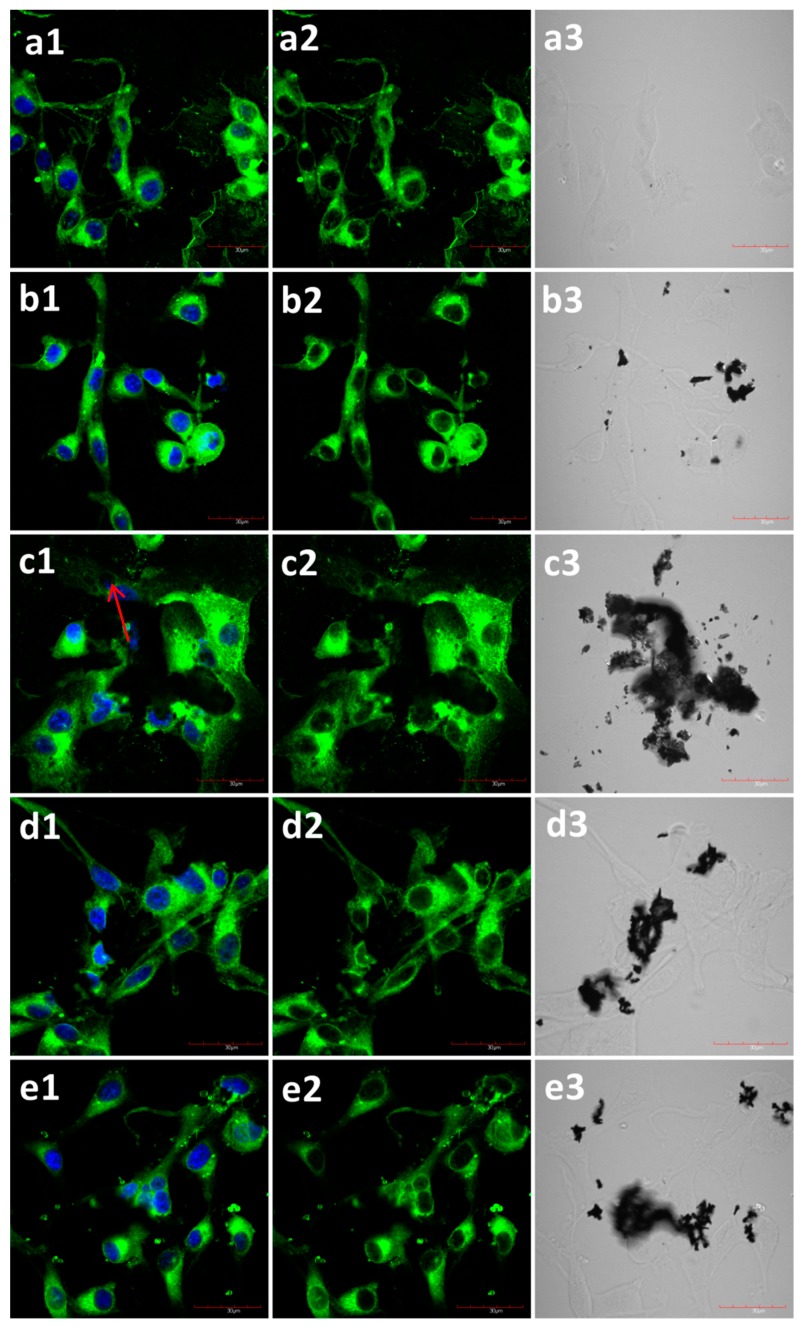
Morphology of untreated U87 cells and those treated with GN and rGO flakes at a concentration of 50 µg/mL after 24 h evaluated by confocal microscopy. Images show the untreated control (**a**) group and groups treated with: GN/ExF (**b**), rGO/Term (**c**), rGO/ATS (**d**), and rGO/TUD (**e**). The pictures marked with the number (**1**) show the combination of 4-Di-10-ASP (green) and DAPI (blue); (2) show the 4-Di-10-ASP (green); and (**3**) show the DIC contrast. The red arrow marks damage to the cell membrane. Scale bar: 30 µm **Abbreviations**: rGO, reduced graphene oxide; GN, graphene; DAPI, 4′,6-diamidino-2-phenylindole; 4-Di-10-ASP, 4-(4-(didecylamino)styryl)-*N*-methylpyridinium iodide.

The morphology of U87 glioblastoma cells treated with GN and rGO flakes was also evaluated by confocal microscopy. Two dyes were used to visualize cell structures. DAPI (Ex = 350, Em = 470 nm) binds selectively to DNA. 4-Di-10-ASP (Ex = 456, Em = 590 nm) is a lipophilic fluorescent dye for marking cell membranes and other hydrophobic structures. Differential interference contrast (DIC) was used to visualize the GN and rGO flakes, enabling visualization of the whole surface of the flake. In the control group, a clearly outlined cell membrane and cell nucleus, as well as numerous connections between cells, were observed (Figure 4a). Cell shape was not retained in any of the treated groups, except in the cells treated with GN/ExF (Figure 4b); in cells treated with GN/ExF, the membrane most closely resembled that of the control cells. All the other treated groups showed disrupted cell membranes. The most extensive damage to the cell membrane was observed in the group treated with rGO/Term (Figure 4c), where the most severe damage to the cell membrane was present (Figure 4c). In each treated group, we observed that graphene adhered to the cell. Using the imaged acquired via DIC, we observed that the graphene flakes adhering to the cells possessed different sizes. In cells treated with rGO/Term (Figure 4c), perforations in the cell membrane were observed by localized reductions in the intensity of the 4-Di-10-ASP signal, which is marked with a red arrow. Duan et al. showed that cells (line A549 and Raw264.7) treated with GO have the light spots clearly corresponding to dark regions under fluorescence-based imaging, suggesting that these features represent holes (pores) in cell membranes [26].

### 2.3. A Balance between Types of Cell Death and Proliferation

**Figure 5:**
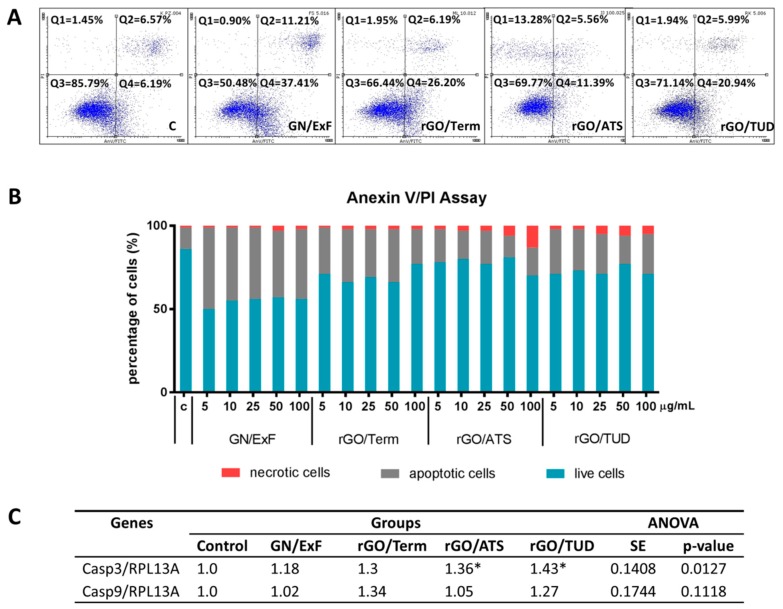
Determining the type of death of U87 cells untreated and treated with GN and rGO flakes via Annexin V-FITC and PI assay, and via the evaluation of *casp3* and *casp9* gene expression. (**A**) Cells were stained with Annexin V/PI and analyzed by flow cytometry. Scatter diagrams show cells untreated and treated with graphene flakes and reduced graphene oxide flakes at the following concentrations: GN/ExF (5 µg/mL), rGO/ATS (100 µg/mL), rGO/Term (10 µg/mL), and rGO/TUD (5 µg/mL, treated for 24 h. Quadrants in the cytograms show live cells (Q3) and individual stages of cell death: Q1—necrotic cells, Q2—late apoptotic cells, and Q4—early apoptotic cells. (**B**) Chart shows the percentage of apoptotic and necrotic cells for all the tested concentrations (5, 10, 25, 50, and 100 µg/mL) of GN and rGOs. (**C**) Gene expression profile in glioblastoma cell line U87; gene expression of *casp3* and *casp9* in U87 cells untreated and treated with graphene (GN) or reduced graphene oxide (rGO) flakes. Bonferroni’s multiple comparison test was used for statistical analysis. Values in rows marked with an asterisk show significant differences. Values marked with one asterisk (*) indicate a *p*-value < 0.005. Values were normalized to that of the reference gene house-keeping gene *rpl13a*. The expression level of the examined genes was expressed as the relative quantitation (RQ) value. **Abbreviations**: PI, propidium iodide; FACS, fluorescence assisted cell sorting; Q, quadrant; C, control group (untreated cells); rGO, reduced graphene oxide; GN, graphene; *casp3*, caspase 3; *casp9*, caspase 9; *rpl13a*, ribosomal protein L13a.

Allotropic forms, including GO [7], rGO [7], GN [8], diamond [34], fullerenes [35], and nanotubes [36], have been studied for many years in the context of inducing apoptosis in normal and cancer cells. The results of these studies have been contradictory, sometimes indicating that GO causes apoptosis [8,35,36] and sometimes showing that it induces proliferation [7,34]. Therefore, in this work, we wanted to examine how flakes that do not contain oxygen, such as flakes of GN, affect glioblastoma cells. We also examined the effect of flakes containing different contents of oxygen, which stems from varying degrees of reduction resulting from using different methods to process the same GO. Our results show that treating glioblastoma cells with flakes of GN and rGOs reduced cellular metabolism. This indicates that the number of viable cells was decreased compared to that of the untreated control group. The decreased metabolic activity indicated that the cells were dying, or dividing slower, or both, which may occur simultaneously. Therefore, we next examined cell death and proliferation. To determine which processes were occurring, we conducted a cytometric assay using Annexin V-FITC (AnnV) and PI. The results of the apoptosis study are shown in Figure 5. The highest percentage of AnnV/PI-positive late-apoptotic cells was observed in the GN/ExF group (Figure 5B). The percentage of apoptotic cells ranged between 40 and 50% for all the tested concentrations of GN/ExF. A small percentage of necrotic cells was also observed in the GN/ExF-treated group. The percentage of apoptotic cells in the remaining groups was less than that observed in the GN/ExF-treated group. In the rGO/Term-treated group (Figure 5B), with rGO/Term concentrations of 5–50 μg/mL, the proportion of apoptotic cells ranged between 28–32%. The highest percentage of necrotic cells, compared with that of the control group, was observed in the rGO/ATS- and rGO/TUD-treated groups (Figure 5B).

Numerous studies have demonstrated that graphene flakes and their derivatives are effective against glioblastoma cells, exerting a tumor suppressive action by activating apoptosis. We have also previously shown that reduced graphene oxide induces apoptosis in both U87 and U118 glioblastoma lines [8]. Moore et al. investigated the impact of nanographene in U-138 glioblastoma cells. The results showed a significant increase in the number of dead cells and decreased the cell density after treatment with graphene at concentrations higher than 50 µg/mL [37]. It was also found that apoptosis is induced in human U251 glioblastoma cells via exposed phosphatidylserine, caspase activation, and fragmentation of DNA [38]. Moreover, graphene and reduced graphene oxides physically damaged cell membranes, increased the permeability of the outer mitochondrial membrane, and disrupted membrane potential [39,40]. Apoptosis can occur via three different pathways. Caspases are usually involved in the process of apoptosis, regardless of the path of initiation. Depending on the stage of apoptosis in which they participate, they are divided into inductive caspases (e.g., caspases 8 and 9) and executive, otherwise called effector, caspases (e.g., caspases 3, 6, and 7) [41]. Therefore, in this study, we analyzed the induction caspase 9 and the effector caspase 3. The results of our real-time PCR analysis indicated that the expression of the *casp9* gene did not show a statistically significant increase in any of the treated cell groups (Figure 5C). A tendency for the increased expression of *casp9* was observed in the rGO/Term and rGO/TUD-treated groups. The level of *casp3* showed a statistically significant increase in the rGO/ATS- and rGO/TUD-treated groups, and similar results were shown in a previous study [7].

**Figure 6:**
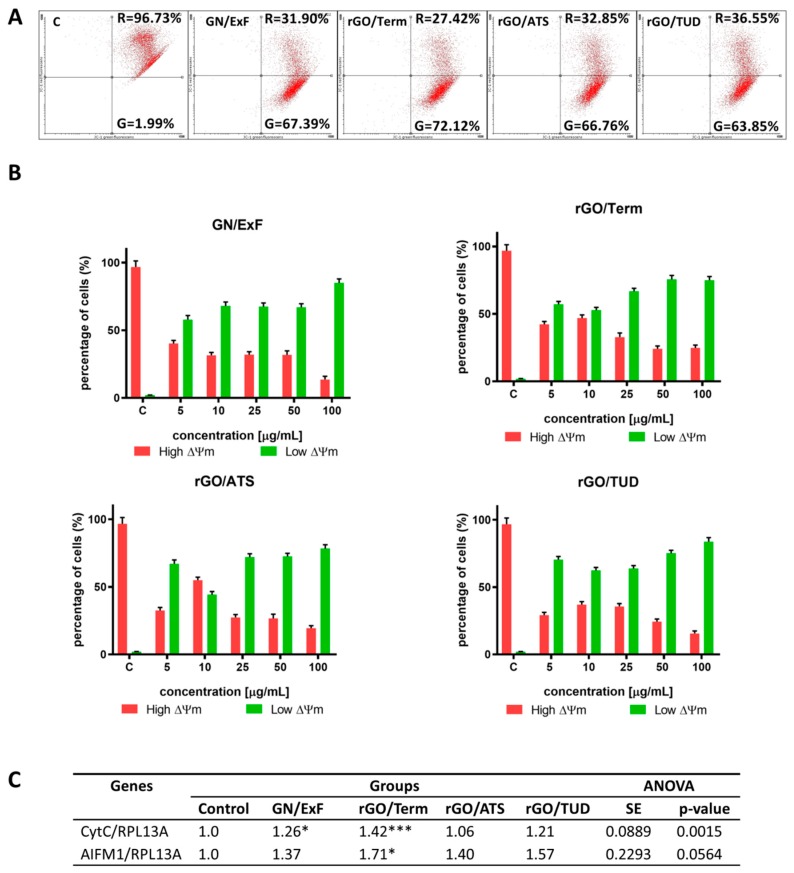
Mitochondrial membrane potential of U87 cells, untreated and treated with GN and rGO flakes, was evaluated using JC-1 dye and the expression of *cytc* and *aifm1* by ΔΔCt method using real-time PCR. (**A**) ΔΨm depolarization was monitored using FACS and JC-1 as markers of mitochondrial membrane potential at 24 h post-exposure to treatment. Cytograms show cells treated with GN and rGO flakes at a concentration of 25 µg/mL. Gated quadrant R (red and green fluorescence) includes cells with intact mitochondrial membranes (high ΔΨm), and quadrant G (green fluorescence) depicts cells with loss of ΔΨm. (**B**) Charts show percentages of cells with high and low ΔΨm for all the tested concentrations (5, 10, 25, 50, and 100 µg/mL) of GN and rGOs. (**C**) The expression of *cytc* and *aifm1* in the glioblastoma cell line U87 untreated and treated with graphene (GN) or reduced graphene oxide (rGO) flakes. Bonferroni’s multiple comparison test was used for statistical analysis. Values in rows marked with an asterisk (*) show statistically significant differences at *p*-value < 0.005. Values marked with one asterisk (*) indicate a *p*-value < 0.005, and three asterisks (***) a *p*-value < 0.001. Values were normalized to that of the reference house-keeping gene *rpl13a*. The expression level of the examined genes was expressed as the relative quantitation (RQ) value. **Abbreviations**: ΔΨm, mitochondrial membrane potential; FACS, fluorescence assisted cell sorting; JC-1, 5,5′,6,6′-Tetrachloro-1,1′,3,3′-tetraethylbenzimidazolylcarbocyanine iodide; C, control group (untreated cells); rGO, reduced graphene oxide; GN, graphene; *cytc*, cytochrome c; *aifm1*, apoptosis inducing factor mitochondrial associated 1; *rpl13a*, ribosomal protein L13a.

Since mitochondria play a key role in apoptosis [41], next we analyzed whether graphene and its derivatives reduce the mitochondrial membrane potential, thereby inducing cell death via the mitochondrial pathway. A JC-1 assay was used to examine the mitochondrial membrane potential in U87 cells untreated and treated with graphene and reduced graphene oxide flakes. JC-1 (5,5′,6,6′-tetrachloro-1,1′,3,3′-tetraethylbenzimidazolylcarocyanine iodide) is a lipophilic, cyanocyanine cationic dye that selectively penetrates the mitochondria and can reversibly alter the emission of red fluorescence to green fluorescence in the case of reduced membrane potential (ΔΨm). Healthy cells have a high membrane potential; in healthy cells, JC-1 selectively accumulates in the mitochondria and forms aggregates that show red fluorescence. In apoptotic cells, JC-1 localizes as a monomer exhibiting green fluorescence [42]. The greatest change in the mitochondrial membrane potential was observed in the group treated with GN/ExF at a concentration of 100 μg/mL. In the groups treated with rGO/TUD and rGO/ATS at a concentration of 5 μg/mL, 70.48 and 67.17% of cells, respectively, showed a low mitochondrial membrane potential compared to the cells in other treatment groups, treated using the same concentration (Figure 6B).

The results of the JC-1 cytometric assay prompted us to examine the expression of genes, namely *aifm*1 and *cytc*, important in the mitochondrial pathway of apoptosis. Apoptosis-inducing agent (AIF) is a pro-apoptotic flavoprotein that participates in caspase-independent cell death. Under physiological conditions, AIF is an FAD-dependent mitochondrial oxidoreductase that plays a role in oxidative phosphorylation. Thus, AIF has a protective function in some cell types. After cellular damage, however, AIF is cleaved by calpains and cathepsins, and moves from the mitochondria to the cytosol and then into the nucleus. In the nucleus, it interacts with DNA and causes the chromatin-independent condensation of caspases and large-scale fragmentation of DNA [43]. Overexpression of AIF promotes peripheral chromatin condensation in the nucleus, DNA fragmentation into 50-kb fragments, translocation of phosphatidylserine in the cell membrane, and a decrease in the mitochondrial membrane potential [44]. The expression of the *aifm1* gene showed a statistically significant increase after treatment with rGO/Term (Figure 6C). In the remaining groups, only a tendency to increase was observed.

In healthy cells, cytochrome c (CytC) is located in the space between the outer and inner mitochondrial membrane, where it acts as an electron carrier in the respiratory chain and interacts with cardiolipin. Pro-apoptotic stimuli may lead to permeabilization of the mitochondrial membrane, which results in cytochrome c molecules being released into the cytosol [45]. In the cytosol, CytC combines with the protein factor Apaf-1 and procaspase 9. This three-element complex is called the “death circle” or the apoptosome. The main task of this complex is the activation of executive caspases (e.g., caspase 3, 6, and 7) [46]. In this study, the expression of the *cytc* gene showed statistically significant increases in the groups treated with GN/ExF and rGO/Term at a concentration of 25 µg/mL (Figure 6C). Limited evidence shows that GO is smaller and less toxic than rGO because it possesses a high oxygen content, smoother edges, and hydrophilic properties [36]. The lowest oxygen content was shown by rGO/Term; this material induced apoptosis more strongly than the rest of the tested compounds. However, the most effective inducer of apoptosis was pure graphene (GN/ExF). This indicates that even a minimal oxygen content decreased the pro-apoptotic activity of graphene and its derivatives. Cells normally die and proliferate. Thus, cellular proliferative potential must also be taken into account in order to check for any compensatory mechanisms. Therefore, we then analyzed the cell cycle and expression of key genes in the regulation of cell cycle and proliferation.

**Figure 7:**
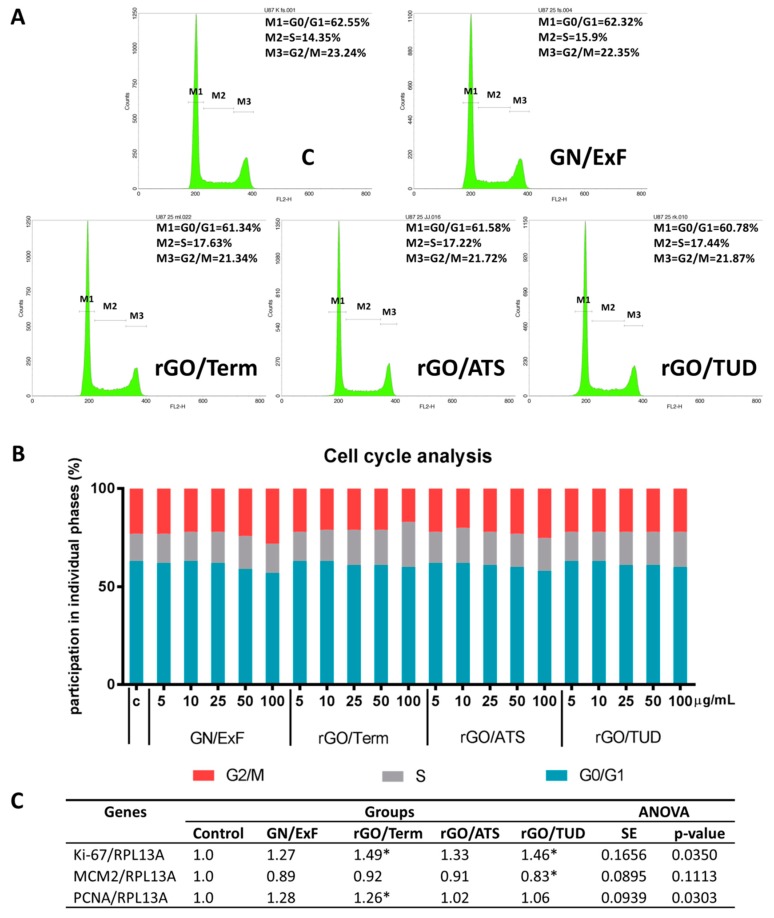
Cell cycle and proliferation in U87 cells untreated and treated with GN and rGO flakes as evaluated by flow cytometry and propidium iodide (PI). (**A**) Histograms were used to analyse the cell cycle in U87 cells untreated and treated with GN or rGO flakes at a concentration of 25 μg/mL. At 24 h post-treatment, cells were stained with propidium iodide after RNase A treatment and DNA content was evaluated by flow cytometry. Histograms show numerical values of individual phases of the cell cycle. M1 = G0/G1 (G0 indicates cells in the resting phase, while G1 indicates production of mRNA and proteins necessary for DNA synthesis); M2 = S (DNA replication phase); M3 = G2/M (G2 indicates preparing for entry into mitosis, and M indicates the mitotic phase). Histograms on the x-axis show the number of cells collected for analysis, while the y-axis shows the intensity of PI fluorescence on a linear scale. (**B**) Chart shows the percentage of cells in individual phases of the cell cycle at all tested concentrations (5, 10, 25, 50, and 100 µg/mL) of GN and rGOs. (**C**) Gene expression profile in glioblastoma cell line U87, untreated and treated with graphene (GN) or reduced graphene oxide (rGO) flakes, was assessed using proliferation markers *ki-67*, *mcm2*, and *pcna*. Bonferroni’s multiple comparison test was used for statistical analysis. Values in rows marked with an asterisk (*) show statistically significant differences at *p*-value < 0.005. Values marked with one asterisk (*) indicate *p*-value < 0.005. Values were normalized to that of the reference hose-keeping gene *rpl13a*. The expression level of the examined genes was expressed as the relative quantitation (RQ) value. **Abbreviations**: C, control group (untreated cells); rGO, reduced graphene oxide; GN, graphene; *ki-67*, nuclear protein detected using monoclonal antibody Ki-67; *mcm2*, minichromosome maintenance complex component 2; *pcna*, proliferating cell nuclear antigen; ANOVA, analysis of variance; *rpl13a*, ribosomal protein L13a; SE, standard error.

For this, we conducted a cytometric analysis of DNA content using propidium iodide, RNase A and analyzed the expression of genes important in proliferation. Cytometric analysis (Figure 7A,B) showed no significant differences in the percentage of cells in the individual phases of the cell cycle. After treatment with rGO/Term at 100 μg/mL, a small increase in the percentage of cells in the S phase of the cycle was observed in comparison with that of the control cells. A small decrease in the percentage of cells in the G2/M phase was observed in the group treated with GN/ExF at a concentration of 100 μg/mL. Kang et al. showed that exposing PC12 cells (a traditional neural target cell line) to rGO at 20 and 50 μg/mL led to cell-cycle arrest in the G0/G1 phase. That study also showed a significant decrease in the number of cells in the G2/M phase, which may be associated with abnormal proliferation and altered mitotic ability [47]. In our study, only a small percentage of cells in the S phase of the cell cycle was observed after treatment with rGOs. These minor changes in the cell cycle prompted us to assess the expression of genes important in proliferation.

We evaluated the expression of *pcna*, *ki-67*, and *mcm2*, which are involved in regulation of the cell cycle and proliferation. Proliferating target nuclear antigen (PCNA) is a cell cycle regulatory protein; its expression increases significantly in the G1 phase, reaching a peak and decreasing in the G2/M phase. PCNA expression shows a periodic change with the replication of DNA in the transition from G1 phase into the S phase [48]. Expression of the Ki-67 protein is also closely related to cell proliferation. During interphase, this antigen is found within the nucleus when the chromosomes are relocated to the surface of the nucleus. The fact that the Ki-67 protein is present during the active cycle (G1, S, G2, and mitosis), but is absent in resting cells (G0), makes it an excellent marker for assessing the growth of a given cell population [49]. MCM2 is one of the minichromosomal maintenance proteins that is necessary for the initiation and regulation of DNA replication. The highest expression of MCM2 occurs in the G1 phase, when it is necessary to initiate DNA synthesis after each turn of the cell cycle. MCM proteins dissociate chromatin irreversibly during the S phase of the cell cycle [50]. The analysis of gene expression showed statistically significant changes (Figure 7). The expression of *pcna* was only significantly increased in the rGO/Term group, whereas *mcm2* expression in the rGO/TUD-treated group was decreased. Sawosz et al. showed a tendency for a decreased expression of *pcna* mRNA in the healthy brain tissue of chicken embryos treated with pure graphene in vivo. This may indicate decreased DNA synthesis during the cell cycle [51]. In this study, the gene expression of *pcna* increased by 26% in the rGO/Term-treated group and by 28% in the GN/ExF-treated group; this was likely due to cell-cycle arrest in the S phase occurring in U87 cells after treatment with GN and rGOs. The *ki-67* gene is highly expressed in glioblastoma and lymphatic metastasis tissues, and is closely related to cell division [52]. Our study is the first to report that treatment with GN and rGO affected the expression of the *ki-67* gene in glioblastoma multiforme cells. So far, there is only one published study describing the effects of GO, functionalized with polyethylene glycol (PEG) and polyethylenimine (PEI), on the cell cycle in five tumor lines (HeLa, HEK293T, A549, HepG2, and MCF-7). The results of that study showed that GO-PEG-PEI nanosheets disrupt the S phase of the cell cycle, leading to a reduction in DNA synthesis [53]. However, our results indicate that cells treated with reduced graphene oxides showed a statistically significant increase in the expression of *ki-67*. The expression of *ki-67* increased by 49% in the rGO/Term-treated group and by 46% in the rGO/TUD-treated group, compared with GN/ExF-treated group, where the expression increased by 27%. The expression of *mcm2* was previously shown to be decreased by treatment with ionizing radiation in the glioblastoma line U251 [54]. In our study, *mcm2* gene expression exhibited a statistically significant decrease of 17% in the rGO/TUD-treated group of U87 cells. This may have occurred because the cell cycle had stopped in the S phase, and the highest expression of *mcm2* was recorded in the G1 phase, when the synthesis of DNA was initiated. In the GN/ExF-treated group, fewer cells were retained in the S phase; therefore, a smaller decrease in *mcm2* gene expression was observed. In rGO/Term-, rGO/ATS-, and rGO/TUD-treated groups, more cells were retained in the S phase; hence, a greater decrease in the expression of *mcm2* was observed.

In summary, the surface chemistry and size of graphene flakes play key roles in the toxicity, transport, and excretion of graphene. Thus, various graphene materials may exert different effects on the body [55]. Jaworski et al. observed that rGOs form aggregates, and flakes smaller than 200 nm gain entry into glioblastoma cells U87 and U118 [7]. Horvath et al. showed that rGO flakes of various sizes can be taken up by cells via endocytosis [56]. Mao et al. suggested that graphene flakes with a larger size and higher degree of oxidation may show a stronger cytotoxicity [57]. This was also confirmed in our study, showing that rGO/TUD, having the largest surface area and highest oxygen content, caused greater cytotoxicity at a concentration of 100 μg/mL than GN/ExF did at the same concentration. We conclude that GN/ExF, in comparison to rGO/ATS and rGO/TUD, showed a stronger tendency to agglomerate within the cell body, indicating that these materials had a stronger affinity for the cells. However, flakes that did not have any oxygen groups, such as those of pure graphene, were the most cytotoxic [8]. We also confirmed that rGOs showed few toxicological effects in comparison with those of GN/ExF.

These results collectively show that the surface and function of graphene play key roles in its physico-chemical properties and in the biocompatibility of various graphene-derived materials. Apoptosis is a coordinated process triggered by three different pathways: the death receptor TNFa (tumor necrosis factor a) and FAS (fragment apoptosis simulating)-mediated extrinsic pathway, located on the cell membrane, and the intrinsic mitochondrial pathway. Theoretically, these pathways may be triggered because rGOs and GN interact with death receptors on the cell membrane. Jaworski et al. showed that entry of rGOs into the cell causes degradation of the mitochondria. rGOs may also interact with surface receptors on the cell membrane, blocking the transport of various substances into the cell, and causing cellular stress and apoptosis [7]. Li et al. suggested that the mitochondrial pathway may be the dominant mechanism underlying apoptosis induced by graphene [58]. Li et al. suggested that GN may alter mitochondrial integrity via a mechanism associated with the activation of pro-apoptotic agents from the Bcl-2 family (Bim, Bax, and Bcl-2). However, when mitochondria are damaged under the influence of rGO, there is no difference in the expression of Bcl-2, indicating that this protein is not involved in the activation of apoptosis [58]. Zhou et al. showed that exposing MDA-MB-231, PC3, and B16F10 cells to graphene platelets led to the direct inhibition of I, II, III, and IV complexes of the Krebs cycle, causing depolarization of the mitochondria and consequent disruption in ATP production [59].

One of the proposed mechanisms underlying the cytotoxicity of graphene involves reactive oxygen species [60,61]; other mechanisms are based on damage to the cell membrane, impaired mitochondrial activity, DNA damage, and interactions with cellular receptors that eventually lead to apoptotic and/or necrotic cell death [21,60,62]. The mechanism involved in the cytotoxicity of graphene and its derivatives is still not fully understood. However, it is assumed, and confirmed by our studies, that flakes of graphene and its derivatives likely induce cell death via the mitochondrial pathway. Therefore, it appears that the mitochondrion is key for the graphene-cell interactions.

## 3. Materials and Methods

### 3.1. Production and Preparation of GN and rGO

Graphene and reduced graphene oxides were supplied by the Institute of Electronic Materials Technology (ITME), Warsaw. Direct graphite exfoliation using Capstone (a fluorinated surfactant) was used to obtain graphene flakes, designated as GN/ExF. Flakes of rGO were produced by reducing previously prepared graphene oxide (GO). GO was obtained by graphite oxidation and exfoliation, according to a modified method by Marcano [63]. Reduced graphene oxide, designated as rGO/ATS, was created by reducing GO with ammonium thiosulphate for 20 h at 95 °C. The molar ratio of the reducing agent to GO was 3:1 and the reduction was conducted at neutral pH. rGO/Term was created by reducing GO powder in an oven at 1000 °C for 1 h under a nitrogen atmosphere. Reduced rGO/TUD graphene oxide was prepared by reducing GO via exposure to thiourea dioxide at 85 °C for 1.5 h. The molar ratio of the reducing agent to GO was 5:1 and pH was set as 9. rGO/ATS and rGO/TUD were purified through pressure filtration on a membrane and then dialysis was used. During these steps, a significant number of residue chemicals were removed from materials; however, there were still some sulfur ions. Then, 1 mg/mL aqueous suspensions were prepared from the GN powder (GN/ExF) and three types of rGO powders (rGO/ATS, rGO/Term, and rGO/TUD). Powders were gently sonicated for 30 min with ultrapure and sterile water. Then, each suspension was diluted to different concentrations, and each dilution was re-sonicated for 15 min immediately prior to being used to treat cells [33]. Appropriate dilutions of GN and rGO flakes (50, 100, 250, 500, and 1000 μg/mL) were prepared for further experiments.

### 3.2. Characterization of GN and rGOs

Elemental analysis and Raman spectroscopy were conducted at the Institute of Electronic Materials Technology (Warsaw, Poland). Elemental analysis of graphene flakes was performed using an O836 oxygen analyzer (LECO, Katowice, Poland) to assess the content of oxygen in the samples. Electrical conductivity of the samples was also examined. The samples were first pressed to form thin pellets (<100 µm). Resistance was determined in Ohm/square (Ω/□) using a Hallotron (ECOPIA HMS 5500) with an external magnetic field of 0.55 T. Raman spectra were collected using a Reinshaw Invia confocal microscope with a 532-nm Nd-YAG laser, under a 100× objective with a 300-nm spot size and 1 mW laser. Measurements of the thickness of GO flakes were conducted with the use of Atomic Force Microscopy (Bruker, Stuttgart, Germany,). Chemical characterization of graphene samples was performed with an FTIR Spectrophotometer (Vertex 80v, Bruker). Zeta potential and size of flakes in water were measured by light scattering using a ZetaSizer Nano ZS model ZEN3500 (Malvern Instruments, Malvern, UK). The shape and size of GN and rGO flakes were examined using a JEM-1220 (JEOL, Tokyo, Japan) transmission electron microscope (TEM) at 80 KeV, and a Morada eleven-megapixel camera (Olympus Soft Imaging Solutions, Münster, Germany). Grids were inserted into the TEM immediately after drying the droplets in dry air.

### 3.3. Cell Cultures

Human glioblastoma U87 and normal Hs-5 cells were obtained from the American Type Culture Collection (Manassas, VA, USA) and maintained in Dulbecco’s Modified Eagle’s culture medium supplemented with 10% fetal bovine serum (Life Technologies, Houston, TX, USA) and 1% antibiotic-antimycotic mixture containing penicillin and streptomycin (Life Technologies). Culture was performed at 37 °C under 5% CO_2_ and at 95% humidity in an INCOMED153 (Memmert GmbH & Co., Germany).

### 3.4. Cell Morphology

U87 cells were seeded at 2 × 10^5^ cells per well in six-well plates and cultured until confluence reached 70–80%. After 24 h, the culture medium was replaced with that containing 10% solutions of GN and rGO flakes at final concentrations of 5, 10, 25, 50, and 100 μg/mL; then, the cells were incubated for another 24 h. The control sample consisted of cells cultured without the addition of any flakes. Cell morphology was evaluated using an inverted light microscope (Leica DMi8, Cairn, Kent, UK) and a confocal microscope (Olympus FV1000, Tokyo, Japan). For confocal microscopy, U87 cell cultures were plated in six-well plates with sterile cover slips placed into each well. After incubation with graphene flakes, the cells were fixed using 4% aqueous paraformaldehyde for 10 min. After fixation, the cells were washed three times with phosphate buffered saline (PBS) without ions. The cells were stained with 4-(4-(didecylamino)styryl)-*N*-methylpyridinium iodide (4-Di-10-ASP; Life Technologies) at 5 mM, and with diamidino-2-phenylindole (DAPI; Sigma, St. Louis, MO, USA) at 1 µg/mL. After each staining, the cells were washed three times with PBS. A drop of Glass Antifade Mountant (Sigma, St. Louis, MO, USA) was applied to each glass slide and slides were stored in the dark. The images were analyzed using two fluorescence channels and differential interference contrast (DIC) on a FV10-ASW 4.2 (Olympus, Tokyo, Japan).

### 3.5. Viability Assays

Cell viability was assessed using an MTT assay. U87 glioblastoma and Hs-5 normal cells were cultured in 96-well plates. Approximately 1.2 × 10^4^ cells were seeded onto each well. After incubating for 24 h, the culture medium was replaced with that containing 10% solutions of the flakes of GN and rGO at the concentrations of 5, 10, 25, 50, and 100 μg/mL. Cells incubated without the addition of any flakes were used as controls. After 24-h incubation, 10 μL MTT (Thermo Fisher Scientific, Waltham, MA, USA) was added to the cultures and incubated for 4 h at 37 °C. After this time, 100 μL of lysis buffer (containing isopropanol, Tween-20, and HCl) was added to the cultures. The lysed cells and dissolved formazan crystals were centrifuged at 440× *g* for 5 min to deposit cell fragments and graphene and rGO flakes used in the test on the bottom of the plate. Then, 100 μL of supernatant was transferred into a new 96-well plate and measured using a spectrophotometer (Infinite M200, Tecan, Durham, NC, USA) at 570 nm excitation and 690 nm emission. The metabolic activity of the cells was expressed as a percentage compared to that of the control. Wells containing only DMEM were used as blanks. We used the formula (ABS_test_ − ABS_blank_)/(ABS_control_ − ABS_blank_), where ABS_test_ is the absorbance of wells exposed to the treatment, ABS_control_ is the absorbance of control wells, and ABS_blank_ is the absorbance of wells without cells [64].

### 3.6. Apoptosis Assay

Apoptosis was evaluated using a FITC Annexin V/Dead Cell Apoptosis Kit (Thermo Scientific, Waltham, MA, USA). U87 cells were seeded at 2 × 10^5^ cells per well in six-well plates and cultured until 70–80% confluence. After 24 h, the culture medium was changed to that containing 10% solutions of the flakes of GN and rGO at the final concentrations of 5, 10, 25, 50, and 100 μg/mL. The control sample consisted of cells cultured without the addition of any flakes. After 24-h incubation with the flakes, the cells were trypsinized, harvested, and washed with cold PBS containing calcium and magnesium ions. After rinsing and centrifugation, each cell pellet was suspended in 100 μL of Annexin binding buffer. Then, 5 μL of FITC (fluorescein isothiocyanate)-labelled Annexin V and 1 μL of propidium iodide were added to each cell suspension. The cells were incubated at 25 °C for 15 min. Then, 400 μL Annexin binding buffer was added to cell suspensions, gently mixed, and stored on ice until introduction into the flow cytometer. Ten thousand events were recorded per sample. Plots were generated using Flowing Software 2.5.1 (Perttu Terho, Turku, Finland). Fluorescence emission intensity was measured using two FL1 channels for FITC at Em = 530 nm and FL2 for PI at Em > 570 nm.

### 3.7. Cell-Cycle Assay

A method based on staining DNA with propidium iodide was used to assess the cell cycle. U87 cells were seeded in six-well plates at 2 × 10^5^ cells per well and cultured until they reached 70–80% confluence. After 24 h, the culture medium was changed to that containing 10% solutions of the flakes of GN and rGO at the final concentrations of 5, 10, 25, 50, and 100 μg/mL. The control sample consisted of cells cultured without the addition of any flakes. After the cells were fixed and harvested, they were rinsed in 5 mL of PBS without calcium or magnesium ions, and then centrifuged at 200× *g* for 5 min at 4 °C. Cells were then suspended in 1 mL of ice-cold PBS. Each cell suspension was gently mixed and slowly dropped into 9 mL of previously chilled 70% ethanol in 15 mL Falcon tubes. The cells were stored for at least 24 h at −20 °C. Then, the cells were centrifuged at 200× *g* for 10 min at 4 °C. The supernatant was carefully removed to leave the remaining loose pellet undisturbed, and the pellets were resuspended in 3 mL of cold PBS, gently mixed, and centrifuged again using the same parameters. Cell pellets were washed three times in 5 mL of cold PBS and centrifugation. The resulting pellet was resuspended in 0.5 mL of staining buffer [containing 10 mL of PBS buffer (*v*/*v*), 0.1% Triton X-100, 2 mg DNase-free RNase A, and 0.4 mL propidium iodide at a concentration of 500 μg/mL]. Cells were suspended in staining buffer, transferred to cytometric tubes, and incubated for 30 min at room temperature in the dark. The samples were filtered using a filter with a 40-μm pore size, and analyzed on a BDFacsCalibur™ cytometer at a flow rate not exceeding 400 objects/s. Analysis was performed within 2 h from the end of incubation. Data were analyzed using a histogram of fluorescence intensity on an FL2 channel and the number of cells introduced. PI was excited at 488 nm with a relatively large Stockes shift, and the emission wavelength was 617 nm.

### 3.8. Analysis of Mitochondrial Membrane Potential

JC-1 dye (Thermo Scientific, Waltham, MA, USA) was used to determine the mitochondrial membrane potential. U87 cells were seeded in six-well plates at 2 × 10^5^ cells per well and cultured until they reached 70–80% confluence. After 24 h, the culture medium was changed to that containing 10% solutions of the flakes of GN and rGO at the final concentrations of 5, 10, 25, 50, and 100 μg/mL. The control sample consisted of cells cultured without the addition of any flakes. The 200 μM solution of JC-1 dye in DMSO was prepared by pre-heating the reagents to room temperature. The cells were suspended in 1 mL of working solution (1 μL/mL) of JC1 and incubated at 37 °C and at 5% CO_2_ for 15 min. The prepared cell suspensions were analyzed in a BDFacsCalibur™ flow cytometer at 488 nm using the FL1 vs. FL2 channels. Plots were generated using Flowing Software 2.5.1 (Perttu Terho, Turku, Finland).

### 3.9. Isolation of Total RNA

For the isolation of total RNA, U87 cells were cultured in 25 cm^2^ culture flasks. Each of the samples, both control and test (treated with flakes GN and rGO), was established in three independent biological repetitions. The solutions of the GN and rGO flakes were added to the cultures at a concentration of 25 μg/mL in a volume not exceeding 10% of the culture volume, and incubated for 24 h. Total RNA was isolated using a PureLink^®^ RNA Mini Kit (Ambion™ Life Technologies, Foster City, CA, USA). The resulting cell pellet was resuspended in lysis buffer containing 1% 2-mercaptoethanol. Then, frozen metal balls were added to the probe and homogenized in a TissueLyser ball mill (Qiagen, Germantown, MD, USA) for 5 min at 50 Hz. The homogenate was centrifuged at 12,000× *g*. The supernatant, containing total RNA, was transferred into a new tube, and one volume 70% ethanol was added into each volume of cell homogenate, following the manufacturer’s instructions. Total RNA was eluted in a volume of 50 μL RNase-free water and stored at −80 °C. The isolated RNA was measured using a NanoDrop 2000 spectrophotometer (Thermo Scientific, Wilmington, DE, USA). cDNA was synthesized with a cDNA High Capacity Reverse Transcription Kit (AppliedBiosystems, Foster City, CA, USA) to reverse-transcript the mRNA to cDNA, using 2200 ng per reaction. The obtained cDNA was measured using a NanoDrop 2000 spectrophotometer and stored for further analysis at −20 °C.

### 3.10. Real-Time PCR

We used the ΔΔCt method to determine the expression of mRNA using real-time PCR: ΔΔCT = ΔCT test sample—ΔCT calibrator sample. The reaction was carried out using 48-well plates and Luminaris Color HiGreen reagents qPCR Master Mix (Thermo Fisher Scientific); 100 ng of cDNA was used for each reaction. The following genes were examined: *casp3*, *casp9*, *cytc*, *aifm1*, *pcna*, *ki-67*, and *mcm2*. The primers used for this procedure are presented in Table 2. *rpl13a* was used as the reference house-keeping gene [65]. The reaction conditions were set as specified by the manufacturer. Each sample was analyzed in duplicate. The procedure was conducted using a StepOnePlus™ Real-Time PCR System.

### 3.11. Statistical Analysis

The data were analyzed with monofactorial analysis of variance using GraphPad Prism 7.04 (GraphPad Software Inc., La Jolla, CA, USA). Differences between groups were tested using Bonferroni’s multiple comparisons test. All mean values are presented using standard deviation or standard error. Differences with *p*-value < 0.05 were considered significant.

## 4. Conclusions

Every type of graphene derivative affects the particular cell type, triggering the activation of a different cellular pathway. Nevertheless, our results indicate that both GN and rGOs can activate the same apoptotic pathway in the U87 glioblastoma cells. The number of oxygen-containing functional groups and the associated number of defects in the carbon crystal structures are the distinguishing features of the investigated forms of graphene. The resistance appeared to be strongly related to the oxygen content in the reduced graphene oxide forms. A higher oxygen content leads to a higher resistance. In the presented study, we also measured other characteristics, like the zeta potential, approximate size of the flakes, and dispersiveness of graphene.

The presence of oxygen groups renders the reduced graphene oxide to be more hydrophilic. The reduced graphene oxides, produced by different methods, showed differences in surface functionalization in the range between 1 to 16%. Among the reduced graphene oxides investigated in the presented study, the oxygen content of rGO/Term (1% O_2_) had the most similar characteristics to the control (GN/ExF). rGOs appeared to be less cytotoxic than GN in U87 glioblastoma cells in vitro. rGO caused cell death mainly via mitochondrial-dependent induction of apoptosis. Our results show that graphene derivatives exerted a scant influence on the cell cycles.

We hypothesized that rGOs has a stronger anticancer impact than GN, which was supported by our results. The cytotoxicity of rGOs may be mainly the result of their direct contact with the glioblastoma cell membrane. An increase in the reduction of oxygen content in rGOs caused an increase in the number of delocalized electrons on the surface of graphene flakes. This may lead to disruption of the signaling pathways in the cell membrane or the direct interaction within cellular structures that are sensitive to electrochemical potential (cell membrane, mitochondria).

In glioblastoma therapy, the most important feature of these flakes is the presence of oxygen-containing functional groups. Oxygen-containing functional groups are involved in the adherence of flakes to the cell body. Our previous studies showed many features of allotropic forms of carbon, which indicated the potential application of that biomaterial in cancer glioma therapy. It was shown, inter alia, that GN, GO, and rGO can induce cell death of the glioma in the process of apoptosis [7,8]. It was also demonstrated that diamond nanoparticles (DN) have excellent anti-angiogenic properties [3,4]. We confirmed that DN, GO, and GN are highly biocompatible and do not exert a significant effect on rat health status within twelve weeks after intraperitoneal administration [66]. Finally, we proved that GN and rGOs cause a strong effect by the decrease of the mitochondrial membrane potential in glioblastoma cells in the way of cell energy center corruption. In this study, for the first time, we confirmed that GN and rGOs can increase the expression of the *ki-67* gene in the glioblastoma cells of the multiform U87 line. Therefore, as continuation of that research, we plan to investigate the impact of GN and rGOs on the mitochondria-dependent energy pathways. The examination of these features will allow for the future application of different graphene forms in clinical trials and may allow for the effective treatment of the malignant brain tumors.

## Figures and Tables

**Table 1:** Physicochemical properties of the GN and rGO flakes.

GN and rGO Flakes	Elemental Analysis (%O)	Resistance (Ohm/square)	Size of Platelets (μm)	Polydispersity Index	Method of Production
GN/ExF	n.a.	0	2.1–3.3	1	exfoliation
rGO/Term	1	n.a.	1.1–2.3	1	thermal
rGO/ATS	3	21	1.9–4.6	1	chemical (ammonium thiosulphate)
rGO/TUD	16	142	3.4–4.7	0.67	chemical (thiourea dioxide)

**Abbreviations**: mV, millivolt; μm, micrometer; rGO, reduced graphene oxide; GN, graphene; ExF, exfoliation; ATS, ammonium thiosulphate; Term, thermal; TUD, thiourea dioxide; n.a., not available.

**Table 2:** Primers used to assess the expression of genes involved in proliferation and type of cell death.

Genes	Forward Primers (5′-3′)	Reverse Primers (5′-3′)
*ki-67*	CCACACTGTGTCGTCGTTTG	CCGTGCGCTTATCCATTCA
*mcm2*	GTGGATAAGGCTCGTCAGAT	GTCGTGGCTGAACTTGTT
*pcna*	GTCTCCTTTCCTCCTCTCTGTT	CCATCCTCAAGAAGGTGTTGG
*casp3*	CAAACTTTTTCAGAGGGGATCG	GCATACTGTTTCAGCATGGCAC
*casp9*	TGCAGGACACACACACAAGAAA	TCCTGGCTAAGACCCCCTT
*cytc*	GCGTGTCCTTGGACTTAGAG	GGCGGCTGTGTAAGAGTATC
*aifm1*	TGCCTGAGCAGAACAAAGGT	CATGCTGCTCACCGTCCTTA
*rpl13a*	CATAGGAAGCTGGGAGCAAG	GCCCTCCAATCAGTCTTCTG

**Abbreviations**: *ki-67*, marker of proliferation *ki-67*; *mcm2*, minichromosomal maintenance protein 2; *pcna*, proliferating target nuclear antigen; *casp3*, caspase 3; *casp9*, caspase 9; *cytc*, cytochrome c; *aifm1*, apoptosis inducing factor mitochondrial associated 1; *rpl13a*, ribosomal protein L13a.
